# BRAIN HEALTH TOGETHER: DEVELOPING AND PILOT-TESTING AN ONLINE GROUP PROGRAM TO REDUCE DEMENTIA RISK

**DOI:** 10.1093/geroni/igad104.1396

**Published:** 2023-12-21

**Authors:** Deborah Barnes, Coles Hoffmann, Margaret Chesney, Henry Brodaty, Linda Chao, Paul Tang, Rebecca Sudore, Patricia Turo

**Affiliations:** University of California, San Francisco, San Francisco, California, United States; Together Senior Health, San Francisco, California, United States; University of California, San Francisco, San Francisco, California, United States; University of New South Wales, Sydney, New South Wales, Australia; University of California, San Francisco, San Francisco, California, United States; Stanford University, Stanford, California, United States; University of California, San Francisco, San Francisco, California, United States; Together Senior Health, San Francisco, California, United States

## Abstract

Growing evidence suggests that multi-domain interventions can slow cognitive decline and reduce dementia risk in older adults. However, most programs have been in-person and/or had relatively long intervention periods (1 to 2-years). The primary goal of this study was to develop and pilot-test Brain Health Together, a 12-week, virtual-group program to reduce dementia risk through targeting modifiable risk factors in older adults who report cognitive decline. Initial content was developed with input from an external Science Advisory Board. 7 individuals who had been participating in online group movement classes for the previous 2 years were invited to pilot-test and provide feedback on Brain Health Together content and structure. In addition to the movement classes, the pilot program included weekly 1-hour group brain health education classes and 30-minute individual brain health coaching sessions for 12 weeks. The first 4 education classes included general content. Based on participant feedback, subsequent classes provided in-depth content on a single brain health risk factor each week, including expert videos and facilitated discussion. Before their first coaching session, participants completed a brief survey about their current lifestyle behaviors and medical conditions related to brain health. This was used to generate an individualized brain health report that summarized areas with low, moderate or high opportunity for change. Coaching sessions focused on setting SMART (Specific, Measurable, Achievable, Realistic, Timely) goals to address modifiable risk factors. After 12 weeks, participants reported more exercise, less loneliness, and longer sleep duration. We are currently enrolling for a larger clinical trial.

